# Applications of Natural Language Processing for the Management of Stroke Disorders: Scoping Review

**DOI:** 10.2196/48693

**Published:** 2023-09-06

**Authors:** Helios De Rosario, Salvador Pitarch-Corresa, Ignacio Pedrosa, Marina Vidal-Pedrós, Beatriz de Otto-López, Helena García-Mieres, Lydia Álvarez-Rodríguez

**Affiliations:** 1 Instituto de Biomecánica de Valencia Universitat Politècnica de València Valencia Spain; 2 CTIC Centro Tecnológico de la Información y la Comunicación Gijón Spain

**Keywords:** stroke, natural language processing, artificial intelligence, scoping review, scoping, review methods, review methodology, NLP, cardiovascular, machine learning, deep learning

## Abstract

**Background:**

Recent advances in natural language processing (NLP) have heightened the interest of the medical community in its application to health care in general, in particular to stroke, a medical emergency of great impact. In this rapidly evolving context, it is necessary to learn and understand the experience already accumulated by the medical and scientific community.

**Objective:**

The aim of this scoping review was to explore the studies conducted in the last 10 years using NLP to assist the management of stroke emergencies so as to gain insight on the state of the art, its main contexts of application, and the software tools that are used.

**Methods:**

Data were extracted from Scopus and Medline through PubMed, using the keywords “natural language processing” and “stroke.” Primary research questions were related to the phases, contexts, and types of textual data used in the studies. Secondary research questions were related to the numerical and statistical methods and the software used to process the data. The extracted data were structured in tables and their relative frequencies were calculated. The relationships between categories were analyzed through multiple correspondence analysis.

**Results:**

Twenty-nine papers were included in the review, with the majority being cohort studies of ischemic stroke published in the last 2 years. The majority of papers focused on the use of NLP to assist in the diagnostic phase, followed by the outcome prognosis, using text data from diagnostic reports and in many cases annotations on medical images. The most frequent approach was based on general machine learning techniques applied to the results of relatively simple NLP methods with the support of ontologies and standard vocabularies. Although smaller in number, there has been an increasing body of studies using deep learning techniques on numerical and vectorized representations of the texts obtained with more sophisticated NLP tools.

**Conclusions:**

Studies focused on NLP applied to stroke show specific trends that can be compared to the more general application of artificial intelligence to stroke. The purpose of using NLP is often to improve processes in a clinical context rather than to assist in the rehabilitation process. The state of the art in NLP is represented by deep learning architectures, among which Bidirectional Encoder Representations from Transformers has been found to be especially widely used in the medical field in general, and for stroke in particular, with an increasing focus on the processing of annotations on medical images.

## Introduction

Stroke, also called “brain attack,” is a medical emergency that occurs when blood flow to a part of the brain is disrupted caused by a clot blocking an artery or by a cerebral hemorrhage due to a ruptured artery. Stroke can result in a range of symptoms and complications depending on the area of the brain that is affected, having impacts on perception, motor control (typically weakness or paralysis on one side of the body, dizziness or difficulty with balance), or behavior (difficulty in speaking or understanding speech), which is a life-threatening emergency that requires immediate medical attention. Although mortality from stroke is decreasing in developed, high-income countries, it remains one of the leading causes of mortality and disability along with ischemic heart disease, and the prevalence of people living with the effects of stroke is increasing due to the growing and aging population [1].

Therefore, the economic and social costs related to the hospitalization, treatment, and recovery of stroke patients are increasing, and there is a growing demand for advanced technologies that can assist in clinical diagnosis, treatment, predictions of clinical events, intervention recommendations, rehabilitation programs, and related factors [2]. For instance, a quick diagnosis and treatment of stroke is crucial as it leads to improved outcomes and prognosis among patients treated within the so-called “golden hour” [3].

In this context, novel approaches that complement and go beyond evidence-based medicine are required. Tools based on artificial intelligence (AI), with their ability to process large amounts of data, have been widely discussed in recent years as one of the proposed approaches to improve the care of stroke, assisting in diagnosis, prognosis, treatment, and prevention [3,4].

AI is an interdisciplinary science with multiple approaches, which in recent years has experienced a significant growth in the fields of machine learning (ML) and deep learning (DL). ML and DL algorithms can learn from data and improve their performance over time without being explicitly programmed, and these methods can deal with very large and complex data sets. DL is considered a recent specialization of ML, which uses artificial neural networks to extract complex representations and features from data. Throughout the manuscript, a distinction is made between DL, used for algorithms based on multilayered neural networks, and traditional ML based on other techniques.

The application of AI to the management of stroke is a topic that has gained a lot of traction in the general field of health informatics [5], partly owing to the remarkable impact of stroke in public health and the subsequent high demand for effective and efficient tools to diagnose and treat stroke. Moreover, the complexity and variety of stroke casuistry make it a good target for AI solutions, which are especially suited to process large amounts of data from a wide range of sources, identify patterns and trends in large data sets, and learn and adapt to new data.

A domain where those advances have produced particularly good results is natural language processing (NLP), which is a promising tool for medicine to unlock the full potential of electronic health records (EHRs), since it might be used to automatically transform clinical text into structured clinical data that can guide clinical decisions [6,7]. The potential of NLP in the analysis of EHR data is particularly appealing given the great quantity of data contained in these records. Notwithstanding their importance, such data are intractable with conventional mathematical methods, since they are recorded in clinical reports, prescriptions, annotations on medical images, and generally unstructured texts [8].

NLP can assist in the identification of patterns and trends in large data sets, which can improve the understanding of factors that contribute to the development of diseases and can in turn help to define more effective prevention and treatment strategies. NLP can also be used in the analysis of particular cases to guide decisions and potentially delay or prevent the onset of the disease. NLP can also be used to develop intelligent systems to find relevant information in the medical literature [9].

Nevertheless, NLP poses particular challenges, including the protection of privacy in the extraction of data, since personal information is often mixed with other data; the variety of the quality and format of EHR data, which depend on the source and software used to collect them; and the difficulty of annotating data samples for training [10]. Therefore, to unlock the potential of NLP in the exploitation of EHRs, researchers and developers need to combine different advanced ML techniques, apply careful data management, and gain a deep understanding of the clinical domain. There is, however, a paucity of guidance on selecting appropriate methods tailored to the health care industry [11].

This scoping review aimed to gather the knowledge that might help in that guidance by investigating how NLP is used to deliver a smarter health care in different phases of stroke disorders (prevention, diagnosis, treatment, and prognosis). The primary questions that served as a guide for the review are: (1) In which phases or contexts of stroke management is NLP used (prevention, diagnosis, treatment, and/or prognosis)? (2) Which are the main benefits of applying NLP to stroke management, related to clinical, social, and economic factors? and (3) What types of clinical data are collected and used by NLP in stroke management (ie, demographic data, medical notes, physical and functional examination, reports of laboratory or medical devices)?

This review also focused on the following secondary questions: (1) What NLP methods, AI algorithms, and tools are used in stroke studies? (2) Which AI techniques or frameworks are used to process and analyze the data? (3) Are there algorithms and NLP software specifically tuned for stroke? and (4) Which tools have the best performance and how do they compare to others?

## Methods

### Design

The unregistered protocol for this review was created following the PRISMA-ScR (Preferred Reporting Items for Systematic reviews and Meta-Analyses extension for Scoping Reviews) guidelines [12] and the JBI Manual for Scoping Reviews [13].

### Inclusion Criteria

The target patient population of this scoping review included adults that had suffered stroke and people at risk of stroke due to a history of predisposing vascular background or other conditions that increase the risk of developing stroke, including mental illness or heart diseases such as a reduced ejection fraction.

The main concept of interest was the use of NLP in stroke management in public or private health care systems, including use cases and the data and technologies involved in those applications. We considered both the application of NLP for monitoring and decision-making of individual patients as well as for the planification of care resources in the management of stroke cases.

We were interested in any context where prevention, treatment, or rehabilitation of stroke might take place, ranging from early detection outside or inside clinical settings, diagnosis and evaluation of cases, clinical decision-making, administration and monitoring of rehabilitation, and postrehabilitation management.

The types of evidence sources taken into account included articles from peer-reviewed journals, books, and conference papers, considering both primary research studies and systematic or scoping reviews, as well as reports from scientific, medical, or government institutions.

### Search Strategy

The search was performed in the electronic databases of Scopus and Medline through PubMed, using the keywords “natural language processing” and “stroke,” restricted to articles published in the last 10 years, between 2013 and 2022.

### Selection Process

The results of the search were imported into the Zotero Reference Manager software (Corporation for Digital Scholarship, Virginia), which was used to filter out duplicate records. Titles and abstracts of the filtered list were screened independently by two reviewers to ascertain their eligibility according to the inclusion criteria. Disagreements were resolved in a discussion session between the reviewers to obtain a consensus.

The full text of the papers was read by two independent reviewers to extract the relevant data as described below. An internal cross-validation by three other experts on the topic was also considered. Works whose content did not meet the eligibility criteria or did not contain sufficient information to answer the primary questions were excluded and those that reported the same results from the same study were treated as duplicates. The record of rejected works was shared by the reviewers to confirm the decisions of either part.

### Data Extraction and Presentation of Results

The reviewers filled out a table with the following data from each work included in the final selection: type of study, primary diagnosis, related diseases that were used either as inclusion criteria or as predictors in the data analysis, sample size (if suitable), and qualitative responses to the primary and secondary questions.

Works were classified depending on whether or not they reported experimental studies, and those that did were further subclassified as clinical trials or different types of observational studies: cross-sectional, retrospective or prospective, and cohort or case-control studies.

A dictionary of terms was defined for the tabulated records of the primary and secondary questions and their relative frequencies were calculated. In addition, the relationships between answers were analyzed in two different multiple correspondence analyses (MCAs), which can be employed to detect and represent underlying structures in categorical data sets (ie, frequent co-occurrence of specific categories in two or more variables) [14]. One of the MCAs focused on the primary questions, seeking relationships between the context of application (eg, classification of diagnostics, prognosis of outcomes) and the types of data that were processed. The other MCA focused on the secondary questions, seeking relationships between NLP methods and software tools. In both analyses, the type of AI models (general ML, DL, or rule-based algorithms) was also included as a variable. The analysis was performed in R [15], using the packages *factoMineR* [16] and *factoextra* [17] for MCA and its graphical representation.

## Results

### General Description of the Studies

**Figure 1:**
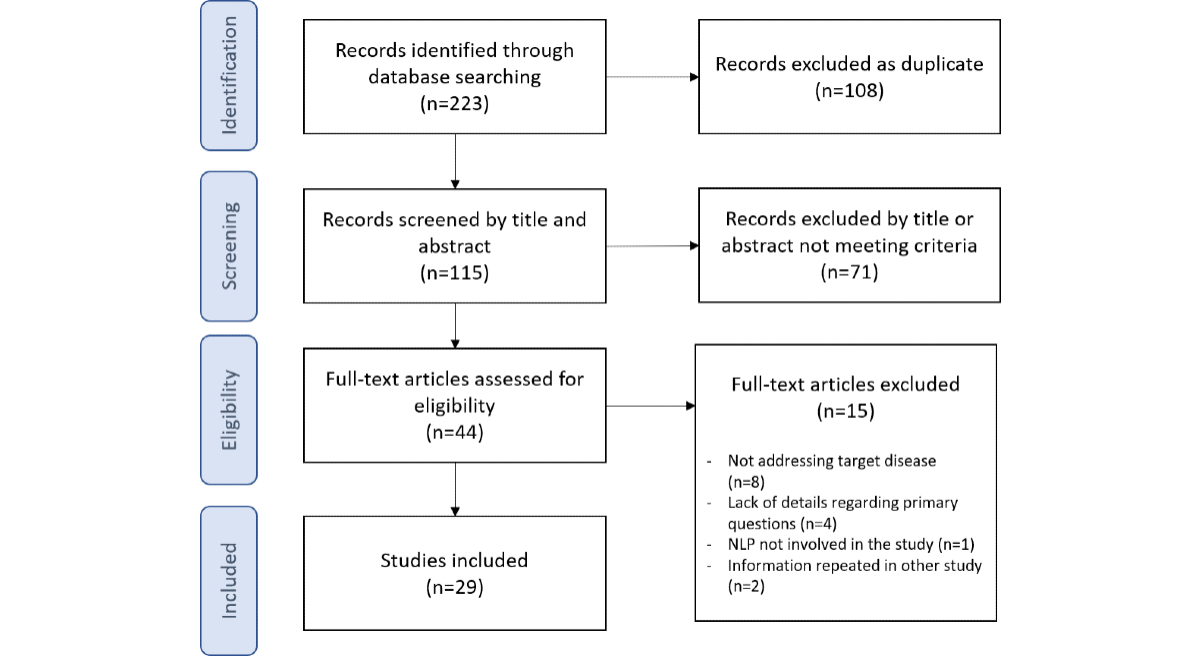
Flow diagram of the review process. NLP: natural language processing.

A total of 115 unique papers were identified out of 223 records obtained in the search; 29 studies were eventually included for data extraction and analysis after screening by title and abstract and reading of the full text (see the flow diagram in Figure 1).

The general characteristics of the 29 reviewed studies (year, type of study, target diseases, and sample size), together with the items extracted from the primary and secondary questions are respectively presented in Tables 1, 2, and 3.

**Table 1:** Summary of the included studies: study type, sample size, type of stroke, and other diseases or conditions taken into account.

Reference	Year	Type of study	Sample size^a^	Type of stroke	Other conditions
Zhao et al [18]	2021	Cohort study	4914	Transient ischemic attack, hemorrhagic stroke	AF^b^
Zanotto et al [19]	2021	Retrospective cross-sectional cohort study	188	Ischemic stroke	AF, CAD^c^, DM^d^, dyslipidemia, hypertension, smoking, other^e^
Sung et al [20]	2022	Retrospective cohort study	3847	Acute ischemic stroke	AF, CHF^f^, DM, cancer, hyperlipidemia, hypertension
Sung et al [21]	2021	Retrospective cohort study	3847	Acute ischemic stroke	AF, CHF, DM, cancer, hyperlipidemia, hypertension
Miller et al [22]	2022	Retrospective cohort study	918	Ischemic stroke	Other
Mayampurath et al [23]	2021	Cohort study	965	Acute ischemic stroke, hemorrhagic stroke	Other
Lineback et al [24]	2021	Retrospective cohort study	2855	Ischemic stroke, hemorrhagic stroke	AF, CAD, CHF, DM, cancer, hyperlipidemia, hypertension, other
Kogan et al [25]	2020	Retrospective cohort study	7149	Ischemic stroke, hemorrhagic stroke, transient ischemic attack	None
Heo et al [26]	2020	Retrospective cohort study	1810	Acute ischemic stroke	DM, dyslipidemia, hyperglycemia, hypertension, smoking, other
Deng et al [27]	2022	Feasibility study	1000 (simulated)	Hemorrhagic stroke	DM, hypertension
Bacchi et al [28]	2019	Cohort study	2201	Transient ischemic attack	None
Yu et al [29]	2021	Cohort study	1320	Ischemic stroke, hemorrhagic stroke	None
Wheater et al [30]	2019	Cohort study	2160	Ischemic stroke, hemorrhagic stroke	None
Sung et al [31]	2020	Cohort study	4640	Acute ischemic stroke	None
Sung et al [32]	2018	Feasibility study	90	Acute ischemic stroke	Hyperglycemia, other
Shek et al [33]	2021	Cohort study	2327	Stroke comorbidities	AF, CHF, DM, hypertension
Rannikmäe et al [34]	2021	Cohort study	207	Intracerebral hemorrhage, subarachnoid hemorrhage, and ischemic stroke	None
Ong et al [35]	2020	Cohort study	721	Acute ischemic stroke	None
Mowery et al [36]	2016	Cohort study	498	Ischemic stroke	CAD, CHF, DM, hypertension
Li et al [37]	2021	Cohort study	3971	Acute or subacute ischemic stroke	None
Leung et al [38]	2021	Cohort study	182	Not applicable	Other
Kim et al [39]	2019	Cohort study	3204	Acute ischemic stroke	None
Kent et al [40]	2021	Retrospective cohort study	261,960	Ischemic stroke	AF, CAD, CHF, DM, hyperlipidemia, hypertension, other
Lin et al [41]	2021	Retrospective cohort study	1700	Acute ischemic stroke	Other
Guan et al [42]	2021	Cohort study	1598	Ischemic stroke	CHF, other
Garg et al [43]	2019	Cohort study	1091	Ischemic stroke	AF, CAD, DM, hyperlipidemia, hypertension
Farran et al [44]	2022	Retrospective cohort study	16,916	Not applicable	AF
Elkin et al [45]	2021	Cohort study	96,681	Not applicable	AF
Bacchi et al [46]	2022	Cohort study	438	Ischemic stroke, hemorrhagic stroke	None

^a^Number of patients involved.

^b^AF: atrial fibrillation.

^c^CAD: coronary artery disease.

^d^DM: diabetes mellitus.

^e^Other refers to conditions that are not already listed in the table.

^f^CHF: coronary heart failure.

The vast majority were cohort studies that analyzed clinical aspects, along with societal or economic aspects of the disease in some cases, at the moment of data gathering. Approximately one third of the papers (n=10) also included a retrospective analysis and 2 of them were limited to feasibility studies. Although the search included a time span of 10 years, only one of the studies included in the review was older than 5 years [36] and most studies (n=19) had been published in the last 2 years (2021 or 2022).

Most studies (n=24) focused on ischemic stroke (either acute, subacute, or transient); the second most frequent type of stroke was hemorrhagic stroke (n=9), which in the majority of cases was in addition to and not excluding ischemic stroke (only 2 papers dealt exclusively with hemorrhagic stroke). Many studies considered other clinical conditions that were used to select the patients or were included as information taken into account by the models. The most common conditions were atrial fibrillation, diabetes mellitus, and hypertension; each of them was considered in one third of the reviewed papers (n=10). Other diseases that were considered with smaller frequency were hyper- or dyslipidemia, hyperglycemia, hypercholesterolemia, coronary heart failure, smoking, or cancer.

The sample size of the cohort studies was highly varied, ranging between 182 patients [38] and more than 260,000 patients [40], with a median sample size of 2160 patients. The two feasibility studies were conducted either with simulated cases [27] or with a smaller sample of 90 patients [32].

Table 4 shows the frequency of each category used to classify the answers to the primary and secondary questions, except for the question about the specificity of algorithms and NLP tools for stroke, since there was little variability in those answers.

**Table 2:** Summary of the answers to the primary questions.

Reference	Context for NLP^a^ use	Expected benefits	Types of clinical data^b^
Zhao et al [18]	Prevention and diagnosis (classification)	CLINICAL: improved triage	Demographic data, laboratory test results, medical history, medication
Zanotto et al [19]	Prognosis (outcomes)	CLINICAL: care information management, characterize patients, prediction of outcomes, risk assessment; SOCIETAL: supporting research studies; ECONOMIC: public health management	Diagnostic reports
Sung et al [20]	Prognosis (outcomes)	CLINICAL: prediction of outcomes	Annotated medical images, clinical scales, demographic data, diagnostic reports, medical history, patient treatments
Sung et al [21]	Prognosis (outcomes)	CLINICAL: prediction of outcomes, risk assessment	Annotated medical images, clinical scales, demographic data, diagnostic reports, functional outcomes data
Miller et al [22]	Prognosis (outcomes)	CLINICAL: prediction of outcomes, risk assessment	Annotated medical images, diagnostic reports
Mayampurath et al [23]	Diagnosis (classification)	CLINICAL: improved triage	Diagnostic reports
Lineback et al [24]	Prognosis (recurrence)	CLINICAL: care information management	Demographic data, diagnostic reports, medical history, medication, patient treatments
Kogan et al [25]	Prognosis (outcomes)	CLINICAL: administration of treatments, care information management, improved triage, prediction of outcomes	Demographic data, clinical scales, medical history, patient treatments, medication
Heo et al [26]	Prognosis (outcomes)	CLINICAL: prediction of outcomes	Annotated medical images, diagnostic reports
Deng et al [27]	Diagnosis (details); treatment	CLINICAL: administration of treatments	Annotated medical images, clinical scales, diagnostic reports, medical history
Bacchi et al [28]	Diagnosis (classification)	CLINICAL: stroke cause prediction	Annotated medical images, diagnostic reports, medical history, medication
Yu et al [29]	Diagnosis (details)	CLINICAL: improved triage; ECONOMIC: public health management	Annotated medical images, diagnostic reports
Wheater et al [30]	Diagnosis (classification)	CLINICAL: disease surveillance, improved triage; ECONOMIC: public health management	Annotated medical images, diagnostic reports
Sung et al [31]	Prevention and diagnosis (classification)	CLINICAL: administration of treatments, care information management, disease surveillance; ECONOMIC: public health management	Diagnostic reports
Sung et al [32]	Diagnosis (details); treatment	CLINICAL: administration of treatments	Diagnostic reports, laboratory test results, medical history
Shek et al [33]	Diagnosis (comorbidities)	CLINICAL: care information management	Demographic data, medical history
Rannikmäe et al [34]	Diagnosis (classification)	CLINICAL: improved triage	Annotated medical images, diagnostic reports
Ong et al [35]	Diagnosis (details)	CLINICAL: administration of treatments, prediction of outcomes; SOCIETAL: supporting research studies	Annotated medical images, diagnostic reports
Mowery et al [36]	Prevention	CLINICAL: risk assessment	Diagnostic reports
Li et al [37]	Diagnosis (classification)	CLINICAL: improved triage	Annotated medical images, diagnostic reports
Leung et al [38]	Diagnosis (details)	CLINICAL: care information management, characterize patients	Annotated medical images, diagnostic reports
Kim et al [39]	Diagnosis (classification)	CLINICAL: care information management, characterize patients	Annotated medical images, laboratory results, demographic data, diagnostic reports, functional outcomes data
Kent et al [40]	Prognosis (outcomes)	CLINICAL: care information management, characterize patients, stroke cause prediction	Annotated medical images, diagnostic reports
Lin et al [41]	Diagnosis (details); prognosis (recurrence)	SOCIETAL: supporting research studies	Diagnostic reports
Guan et al [42]	Diagnosis (classification)	CLINICAL: improved triage	Clinical scales, diagnostic reports
Garg et al [43]	Diagnosis (classification)	CLINICAL: improved triage, risk assessment	Annotated medical images, diagnostic reports, medical history
Farran et al [44]	Diagnosis (classification); prognosis (outcomes)	CLINICAL: stroke cause prediction, disease surveillance; ECONOMIC: public health management	Clinical scales, demographic data, medical history, patient treatments
Elkin et al [45]	Diagnosis (classification)	Not applicable	Clinical scales, demographic data
Bacchi et al [46]	Diagnosis (classification)	Not applicable	diagnostic reports, patient treatment

^a^NLP: natural language processing.

^b^See Multimedia Appendix 1 for the definitions of clinical data types, following Jiang et al [6].

**Table 3:** Summary of the answers to the secondary questions.

Reference	AI^a^ technique	NLP^b^ methods^c^	Other statistical methods^c^	Software packages^c,d^	Performance metrics^c^	Best performing methods
Zhao et al [18]	ML^e^	Regular expressions	LR^f^, RF^g^	MedTagger, Weka	PPV^h^, NPV^i^, F1, sensitivity	RF
Zanotto et al [19]	ML	Ontologies (OWL^j^), BERT^k^, BOW^l^, TF-IDF^m^	CNN^n^, K-NN^o^, RF, SVM^p^, naïve Bayes	spaCy	PPV, F1, sensitivity	SVM ontological rules
Sung et al [20]	ML	Negation extraction ontologies (UMLS^q^)	Gradient boosting	Jazzy spell checker, MetaMap, XGBoost^r^	AUC^s^, IDI^t^, NRI^u^	Not applicable
Sung et al [21]	DL	BOW, BERT (ClinicalBERT)	Not applicable	Jazzy spell checker	AUC, IDI, NRI	Not applicable
Miller et al [22]	DL rule-based	BOW, negation extraction, TF-IDF, BERT (BioClinicalBERT)	LASSO^v^, K-NN, RF, MLP^w^	scikit-learn	AUC, PPV, sensitivity, specificity	BioClinicalBERT (except for rare and continuous outcomes)
Mayampurath et al [23]	ML	N-grams (1- or 2-)	SVM	Not applicable	AUC, PPV, NPV, sensitivity, specificity	Not applicable
Lineback et al [24]	ML	N-grams (1- or 2-), TF-IDF, Word-embedding (Word2Vec)	LASSO, LR, PCA^x^, RF, SVM, gradient boosting, naïve Bayes	XGBoost	AUC	ML methods in general
Kogan et al [25]	ML rule-based	Not applicable	RF, gradient boosting, MLP	Not applicable	Correlations, RMSE^y^	Not applicable
Heo et al [26]	DL	BOW, Word-embedding (sent2vec, BioWordVec)	Decision trees, CNN, LASSO, LSTM^z^, MLP, RF, SVM	Quanteda, NLTK^aa^, Tensorflow, Keras	AUC	Document-level methods, CNN
Deng et al [27]	DL rule-based	BERT	Not applicable	Not applicable	AUC, PPV, NPV, sensitivity, specificity	Not applicable
Bacchi et al [28]	DL	BOW, negation extraction	Decision trees, CNN, LSTM, RF	Not applicable	AUC, PPV, NPV, sensitivity, specificity	CNN
Yu et al [29]	Rule-based	Regular expressions	Not applicable	CHARTextract	PPV, NPV, accuracy, sensitivity, specificity	Not applicable
Wheater et al [30]	Rule-based	Regular expressions, grammatical analysis, ontologies (custom), negation extraction	Not applicable	BRAT rapid annotation tool	PPV, sensitivity, specificity	Not applicable
Sung et al [31]	ML rule-based	Grammatical analysis (part-of-speech), negation extraction, ontologies (UMLS)	Decision trees (CART^bb^), K-NN, LR, RF, SVM	Google spell checker, MetaMap, Weka	Accuracy, κ	Mixed results
Sung et al [32]	Not applicable	Grammatical analysis (part-of-speech), negation extraction, ontologies (UMLS)	Not applicable	Google spell checker, MetaMap, Stata	NPV, F1, sensitivity, specificity	Document-level methods
Shek et al [33]	DL	Grammatical analysis, Negation extraction, Ontologies (SNOMED^cc^)	Not applicable	MedCAT	NPV, F1, sensitivity, specificity	Not applicable
Rannikmäe et al [34]	ML rule-based	Ontologies (UMLS)	Not applicable	SemEHR	PPV, sensitivity	Mixed results
Ong et al [35]	DL	BOW, TF-IDF, Word-embedding (GloVE^dd^)	Decision trees (CART), K-NN, LR, LSTM, RF	scikit-learn, Tensorflow	AUC, F1, accuracy, sensitivity, specificity	GloVE + LSTM
Mowery et al [36]	Rule-based	Regular expressions	Not applicable	pyConTexT	PPV, NPV, sensitivity, specificity	Not applicable
Li et al [37]	ML	BOW, N-gram (2- and 3-), negation extraction	RF	scikit-learn, NLTK	F1, accuracy	Not applicable
Leung et al [38]	DL rule-based	Not applicable	Not applicable	MedTagger	PPV, NPV, accuracy, sensitivity, specificity	Not applicable
Kim et al [39]	ML	N-gram (1- and 2-), TF-IDF	Decision trees, LR, naïve Bayes, RF, SVM	Quanteda	AUC, F1	Single decision trees
Kent et al [40]	DL rule-based	Ontologies (named entity recognition)	Not applicable	MedTagger	PPV, NPV, accuracy, sensitivity, specificity	Not applicable
Lin et al [41]	DL	BERT (ClinicalBERT, StrokeBERT)	Not applicable	spaCy	AUC, F1	StrokeBERT
Guan et al [42]	ML	Regular expressions, negation extraction	Decision trees (CART), K-NN, LR, RF, SVM	Quanteda	AUC, PPV, NPV, F1, accuracy, specificity	RF
Garg et al [43]	ML	BOW, N-grams (1- to 3-)	Decision trees, K-NN, stacking LR, PCA, RF, SVM, gradient boosting	cTAKES, spaCy, XGBoost	AUC, sensitivity, κ	Stacking, LR, gradient boost
Farran et al [44]	ML	Ontologies (SNOMED), negation extraction	Not applicable	MedCAT	Accuracy	Not applicable
Elkin et al [45]	ML	Ontologies (SNOMED)	Not applicable	HD-NLP^ee^	PPV, NPV, sensitivity, specificity	Not applicable
Bacchi et al [46]	ML	BOW, N-grams (1- to 3-), negation extraction	Decision trees, LR, RF	scikit-learn, NLTK	AUC, PPN, NPP, sensitivity, specificity	RF

^a^AI: artificial intelligence.

^b^NLP: natural language processing.

^c^See brief descriptions of the NLP tools, statistical methods, software packages, and performance metrics in Multimedia Appendix 2 [47-51].

^d^Excluding general programming frameworks like Python or R.

^e^ML: machine learning.

^f^LR: logistic regression.

^g^RF: random forest.

^h^PPV: positive predictive value.

^i^NPV: negative predictive value.

^j^OWL: Web Ontology Language.

^k^BERT: Bidirectional Encoder Representations from Transformers.

^l^BOW: bag-of-words.

^m^TF-IDF: term frequency-inverse document frequency.

^n^CNN: convolutional neural network.

^o^K-NN: K-nearest neighbor.

^p^SVM: support vector machine.

^q^UMLS: Unified Medical Language System.

^r^XGBoost: extreme gradient boosting.

^s^AUC: area under the curve.

^t^IDI: integrated discrimination index.

^u^NRI: Net Reclassification Index.

^v^LASSO: least absolute shrinkage and selection operator.

^w^MLP: multilayer perceptron.

^x^PCA: principal component analysis.

^y^RMSE: root mean squared error.

^z^LSTM: long short-term memory.

^aa^NLTK: Natural Language Processing toolkit for Python.

^bb^CART: classification and regression tree.

^cc^SNOMED: Systematized Nomenclature of Medicine.

^dd^GLoVE: Global Vectors for Word Representation.

^ee^HD-NLP: high-definition natural language processing.

**Table 4:** Frequencies of distinctive items found in primary and secondary questions among the included studies (N=29).^a^

Variable and category^b^			Studies, n (%)
**Context**			
	Diagnostic (classification)	13 (45)	
	Diagnostic (details)	6 (21)	
	Prognostic (outcomes)	8 (28)	
	Prognostic (recurrence)	2 (7)	
	Prevention	3 (10)	
	Treatment	2 (7)	
**Clinical benefits**			
	Improved triage	9 (31)	
	Care information management	8 (28)	
	Prediction of outcomes	7 (24)	
	Administration of treatments	5 (17)	
	Risk assessment	5 (17)	
	Patient characterization	4 (14)	
	Disease surveillance	3 (10)	
	Stroke causes	3 (10)	
**Data sources**			
	Diagnostic reports	24 (83)	
	Annotated images	15 (52)	
	Medical history	10 (34)	
	Demographic data	9 (31)	
	Clinical scales	7 (24)	
	Treatments	5 (17)	
	Medication	4 (14)	
	Laboratory results	3 (10)	
	Functional outcomes data	2 (7)	
**Artificial intelligence technique**			
	ML^c^	15 (52)	
	DL^d^	10 (34)	
	Rule-based	10 (34)	
**Natural language processing tools**			
	Negation extraction (NEGEX)	11 (38)	
	Ontologies	10 (34)	
	Bag-of-words (BOW)		
	*n*-grams	6 (21)	
	Bidirectional Encoder Representations from Transformers (BERT)	5 (17)	
	Regular expressions (REG-EXPR)	5 (17)	
	TF-IDF^e^	5 (17)	
	Grammatical analysis	4 (14)	
	Word-embedding	3 (10)	
**Other statistical tools**			
	Random forest (RF)	14 (48)	
	Decision trees	8 (28)	
	Support vector machine (SVM)	7 (24)	
	Logistic regression (LR)	7 (24)	
	K-nearest neighbor (K-NN)	6 (21)	
	Gradient boosting	4 (14)	
	Naïve Bayes	3 (10)	
	Multilayer perceptron (MLP)	3 (10)	
	Long short-term memory (LSTM)	3 (10)	
	Principal component analysis (PCA)	2 (7)	
**Software packages**			
	scikit-learn	4 (14)	
	NLTK^f^	3 (10)	
	spaCy	3 (10)	
	Quanteda	3 (10)	
	MedTagger	3 (10)	
	MetaMap	3 (10)	
	XGBoost^g^	3 (10)	
	MedCAT	2 (7)	
	Weka	2 (7)	
	Tensorflow	2 (7)	
**Performance metrics**			
	Based on ratios (PPV^h^, NPV^i^, F1, accuracy, sensitivity, or specificity)	23 (79)	
	Based on ROC^j^ curves (AUC^k^, C-statistic)	14 (48)	
	Differential measures (NRI^l^, IDI^m^)	2 (7)	

^a^Only the items that occurred more than once are reported in this table; however, since different items often overlapped in each study, the frequencies of each variable normally sum to more than 100%.

^b^See brief descriptions of the NLP tools, statistical methods, software packages, and performance metrics in Multimedia Appendix 2 [47-51].

^c^ML: machine learning.

^d^DL: deep learning.

^e^TF-IDF: term frequency-inverse document frequency.

^f^NLTK: Natural Language Processing toolkit for Python.

^g^XGBoost: extreme gradient boosting.

^h^PPV: positive predictive value.

^i^NPV: negative predictive value.

^j^ROC: receiver operating characteristic.

^k^AUC: area under the curve.

^l^NRI: Net Reclassification Index.

^m^IDI: integrated discrimination index.

The most frequent context of stroke in which the studies were applied was the diagnostic phase, followed by the prognosis of outcomes. The potential benefit of the results on clinical processes (eg, improving the triage of patients depending on the type or severity of stroke, more efficient management of care information) was the main focus of all studies but one [41], which chiefly focused on the societal aspect of supporting research studies, similar to two other studies that also evaluated that aspect along with clinical applications. Five of the 29 studies (17%) also considered the potential economic benefit of NLP, in terms of reducing the costs of stroke for the public health sector.

The most frequent source of data for NLP models was diagnostic reports (n=24), followed in many cases by annotations on medical images such as radiographs and scans (n=15). General ML models were used more frequently than DL or rule-based algorithms to process the data (n=15 for ML vs n=10 papers for either DL or rule-based techniques). NLP tools, other statistical methods, and the software packages that were used to implement them highly varied across papers, although there were some associations with the AI technique and other variables (see the next subsection).

In nearly all studies, the AI architectures and algorithms had been adapted to deal with stroke-related data, except for one study that used an ML model for patients with severe mental illness at risk of stroke [44]. One of the studies actually used a software tool that was specifically designed for stroke [41], StrokeBERT, which is a language representation model based on Google’s Bidirectional Encoder Representations from Transformers (BERT) [47]. Other studies used models that were adapted to broader medical terminology, including ClinicalBERT [52], BioClinicalBERT [53], and BioWordVec [54], or models tuned with standard medical vocabularies such as Systematized Nomenclature of Medicine (SNOMED) [55] or Unified Medical Language System (UMLS) [56].

The methods used to compare the performance of the models were also highly varied, although in the greatest majority of cases (n=23) they were metrics based on the ratios of true/false-positive or -negative values (positive predictive value, negative predictive value, sensitivity, specificity, F1 score, or accuracy), and many were based on the receiver operating characteristic curve (n=14); a few studies (n=2) also used measures of classification improvements such as the net reclassification index and the integrated discrimination index [48], and only one study used other statistics such as correlation coefficients or the root mean squared error [25].

Owing to the variety of methods and tools used in the studies, there were few coincidences in the selection of the best ones. The only methods that were chosen as the best performing in more than one study were random forest (n=3), convolutional neural network (n=2), and BERT (n=2).

### Multiple Correspondence Analysis

**Figure 2:**
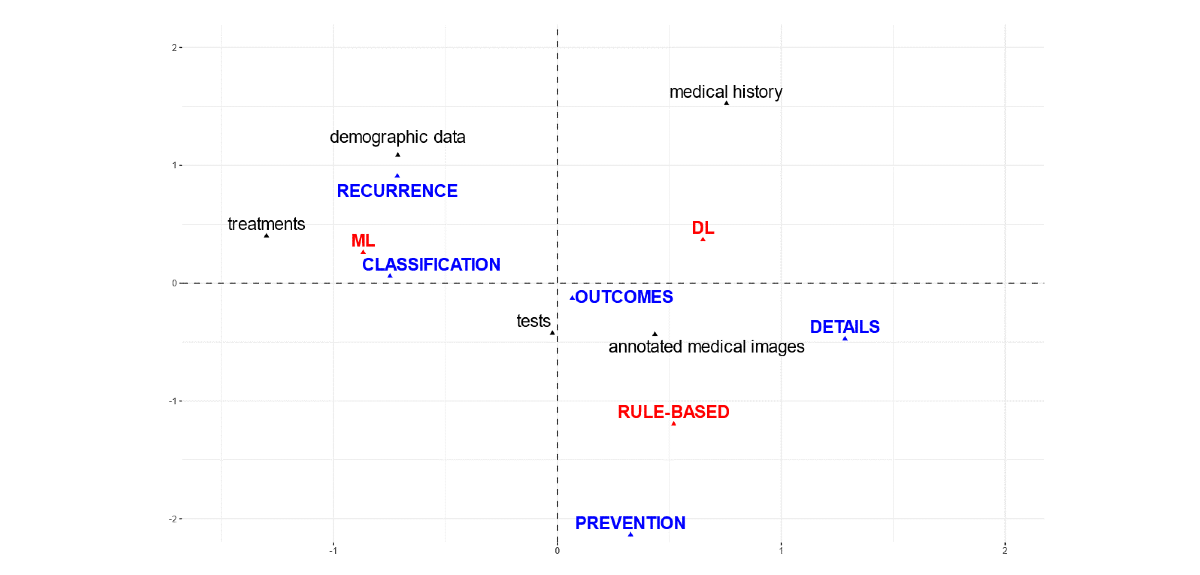
Projection of the scores of the categories in the first two dimensions of the multiple correspondence analysis plot involving context of application, data sources, and artificial intelligence technique. DL: deep learning; ML: machine learning.

**Figure 3:**
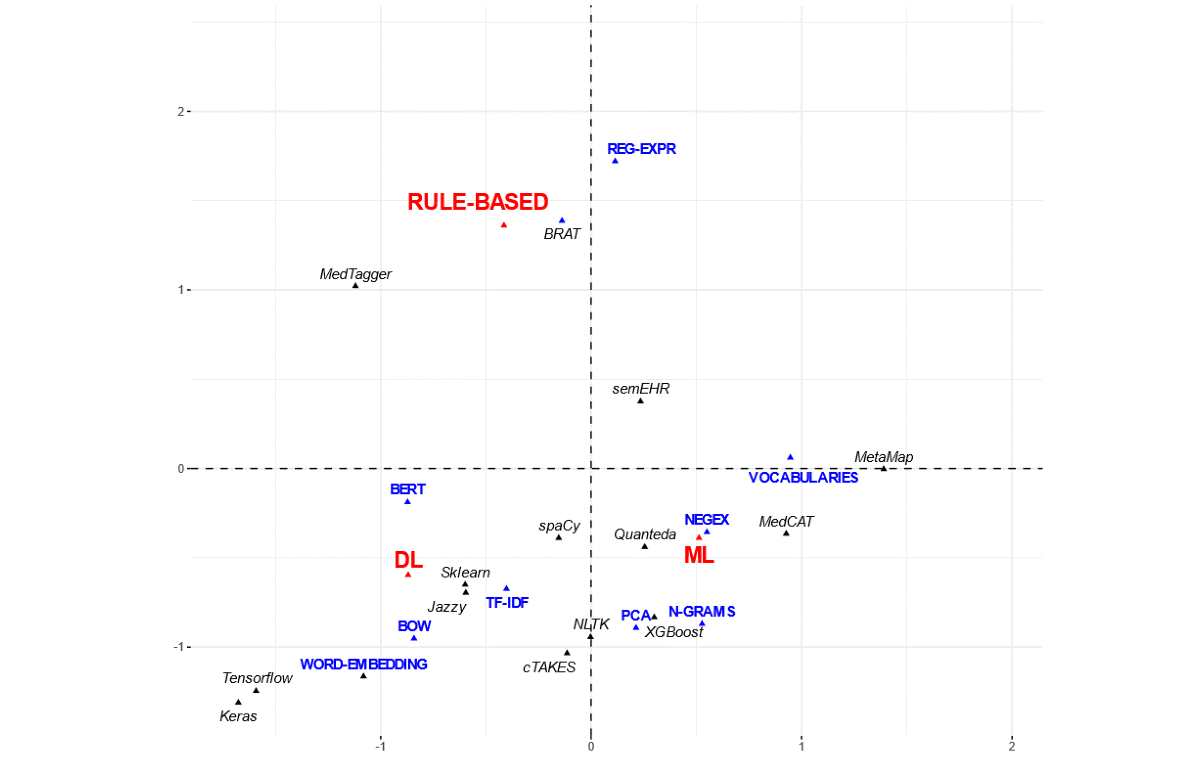
Projection of the scores of the categories in the first two dimensions of the multiple correspondence analysis plot involving natural language processing methods, software, and artificial intelligence techniques. See brief descriptions of the methods and software in Multimedia Appendix 2. BERT: Bidirectional Encoder Representations from Transformers; BOW: Bag-of-words; BRAT: Browser-based Rapid Annotation Tool; DL: deep learning; ML: machine learning; NEGEX: Negation extraction; NLTK: Natural Language Processing toolkit for Python; REG-EXPR; regular expressions; TF-IDF; term frequency-inverse document frequency; XGBoost: extreme gradient boosting.

Figures 2 and 3 show the proximity of the categories that exhibited the closest relationships in the two first dimensions obtained in the MCA.

The common variable used in the analysis (AI technique) was clearly distinguished in the first two dimensions of the MCA plot, which on the one hand separated rule-based techniques from ML and DL and on the other hand separated general ML from DL.

In the first MCA (Figure 2), it could be observed that the studies focusing on the classification of diagnostics (often used for the triage of patients) and prospects of recurrent stroke were often those that also used ML techniques with demographic data and information on treatments. Although the other categories were less tightly related, the text associated with clinical tests and the annotations on images were related more closely to prognostics of outcomes than to other contexts of application, with annotated images also being used to ascertain details of the stroke episode. Both types of studies were frequently approached by DL and sometimes by rule-based techniques.

In the other MCA (Figure 3), AI techniques were separated between ML, DL, and rule-based methods in the two main dimensions of the projected space, although only general ML and DL were closely related to other items.

ML was related to NLP methods that are used in the first steps of the processing pipeline, such as the extraction of text tokens in the form of *n-*grams, detection of negated terms, and use of standard vocabularies. This was mostly performed with software tools such as MetaMap, MedCAT, Quanteda, and extreme gradient boosting.

Conversely, DL was more associated with the usage of BERT, a language representation model based on transformers [47], and NLP methods applied to numerical and vectorized representations of the language tokens, such as the “bag-of-words,” term frequency-inverse document frequency word embeddings, and other word embeddings. This was chiefly performed with software packages such as Tensorflow through Keras and scikit-learn. Other software packages that are often used for NLP, such as Natural Language Processing toolkit for Python, were observed in the middle of the primary axis of the MCA plot, halfway between the general ML and DL architectures.

## Discussion

The research on AI for stroke management has gained greater interest and impact in the last few years [5], and the growing rate of publications found in this scoping review reveals that the same trend is occurring in research on NLP, which is a particular field of AI, applied to the same clinical condition. However, in other aspects, the studies focused on NLP show their own specific trends.

Although the search for this scoping review was very broad, and did not limit the type and phase of stroke to be studied, the vast majority of studies were focused on ischemic stroke in its acute, subacute, or transient stage, and the purpose of using NLP was to improve processes in a clinical context. This focus on clinical contexts is related to the relevance that is attributed to the unstructured information contained in EHRs, (ie, in notes, reports, and annotated images) as predictors of outcomes and complications, which are crucial for proper decision-making, together with the difficulty of processing that information automatically with traditional tools. The deployment of NLP models integrated in the pipelines of an EHR, programmed to automatically ingest and process incoming records [57], or even the patients’ commentaries in emergency through voice-to-text [58], may be used to identify patients at high risk and requiring prompt access to specific treatments; find signs to anticipate impending stroke; or evaluate its severity, type, and risks of complications.

Efficient triage of patients in emergency and early consultations, more accurate diagnostics, or prognostics of outcomes and recurrence were the main intended applications of NLP models in the reviewed studies. Accordingly, the main sources of information exploited by NLP algorithms were clinical data of the patients obtained from their history, especially the diagnostic reports of the current stroke episode. Administration and monitoring of rehabilitation, or postrehabilitation management, were not dealt with in the final selection of studies that were the object of the review.

NLP is itself a broad concept, which involves many types of computational techniques. In its more general sense, NLP comprises all methods and tools that can be used to analyze texts in order to represent human languages, based either on theory of language constructs, semantic mappings, or emulation of linguistic processes occurring in the human brain [59]. The relationships between these tools, types of statistical and ML models, data sources, and applications found by the MCA help to understand how each subset of techniques can be used to solve different problems, and can also help to interpret some trends in the evolution of this technology applied to the clinical management of stroke.

Some of these methods rely on text-processing algorithms that use predefined rules and vocabularies, such as the tokenization of long texts into smaller items, categorization of those items in parts of speech, and construction of syntactic structures, and they have been widely used since long before the recent revolution of big data and DL fields. What this revolution has provided to the field of NLP is the maturity of more complex representations of language data, such as the word embeddings into large-dimensional numeric vectors and their effective processing through deep neural networks, as well as the exploitation of huge databases of texts, such as the Common Crawl data set that includes petabytes of text data, crawled monthly from dozens of billions of web pages [60].

In this context, the state of the art in NLP is represented by DL architectures such as GPT, XLNet, or BERT [61]. Among these, BERT has been found to be particularly widely used in the medical field in general, and for stroke in particular, along with specialized versions fitted to these applications that improve their performance [22,41]. More basic ML algorithms and hybrid approaches with rule-based techniques are still more present than advanced DL networks in the recent research on NLP for stroke, and in some cases, tailored rule-based systems outperformed BERT and its derivatives [19,22]. Support vector machine methods were also found to perform better than BERT in one study [19], although random forest was reported to have the best performance more frequently than any other ML method in the set of reviewed studies [18,42,46]. Some of these results may seem unexpected, given the remarkable performance of DL in general, and particularly large language models (LLMs), in other areas. However, the computational complexity and large data sets needed to train LLMs can limit their current scalability, not outperforming other ML methods that work better on limited training data such as the data sets of the mentioned studies.

The prevalence of studies based on traditional ML methods over those that use DL neural networks may be partly due to the recency of the more complex DL architectures, as well as to the need of larger sets of data to train those models, which raises the bar to conduct studies with that approach. However, it is also interesting to observe that the choice of the AI technique also relates to the type of data that are processed and the context of application of NLP, such that DL is more closely related to studies that involve medical imaging with annotations to prognosticate the outcomes of stroke.

Taking into account these pieces of evidence, and considering the future of NLP in stroke, further development of LLMs in the biomedical field may be expected. LLMs emerged in 2018 as a class of language models that use neural networks with billions of parameters trained on huge amounts of unlabeled text data through self-supervised learning. LLMs are often based on transformers, a self-attention mechanism to compute contextual relationships between the input tokens [62]. However, innovation in the NLP field will come from the development of these models for medical specialties such as stroke. These biomedical LLMs can be trained not only with data sources from EHRs but also from scientific and clinical publications and social network posts from specialized fields. The particularity is that these models need to be trained on much larger databases than those used by classical ML algorithms to achieve adequate performance metrics. This involves combining computational resources and very large data sources, an option that is not always available for the existing resources in research.

## Appendix

Multimedia Appendix 1 Categories of clinical data.

Multimedia Appendix 2 Description of artificial intelligence (AI), natural language processing (NLP), and statistical tools.

Multimedia Appendix 3 PRISMA-ScR checklist.

## Abbreviations

AI: artificial intelligence
BERT: Bidirectional Encoder Representations from Transformers
DL: deep learning
EHR: electronic health record
LLM: large language model
MCA: multiple correspondence analysis
ML: machine learning
NLP: natural language processing
PRISMA-ScR: Preferred Reporting Items for Systematic reviews and Meta-Analyses extension for Scoping Reviews
SNOMED: Systematized Nomenclature of Medicine
UMLS: Unified Medical Language System
