# Supplementary material for: Applications of Natural Language Processing for the Management of Stroke Disorders: Scoping Review
Source: JMIR Med Inform. 2023 Sep 6;11:e48693. doi: 10.2196/48693 (PMC10512117; doi:10.2196/48693)
Supplement: Multimedia Appendix 1 [file medinform_v11i1e48693_app1.docx]

**Appendix 1: Categories of clinical data**

The categories included in the following table classify different types of clinical data related to the management of stroke, based on Jiang et al [6].

***Demographic data***: This item includes information associated to the patients’ age, sex, race, ethnicity, insurance status, marriage status, smoking habits, etc.

***Medical notes:***

1. **medical history**: history of current and past illness, clinical consultations, health habits, etc.
2. **diagnostic reports**: type of stroke (ischemic, hemorrhagic/ acute, transient), and clinical findings related to the stroke episode, such as motor weakness, sensory deficit, speech dysfunction, visual field defect, disorder of eye movement, or impaired coordination.
3. **medication**: information about medications taken during the process and/or discharge.
4. **patient treatments**: procedures followed during the stroke process.

***Physical and functional examination****:*

1. **clinical scales:** information related to pain and/or disability experienced by the patient, measured by published scales.
2. **functional outcomes data**: measurements of mobility, strength or coordination, made during the physical examination of the patient.

***Laboratory or medical devices reports***

1. ***annotated medical images***: text integrated on medical images, through annotations obtained from medical devices for radiographs, computed tomography angiography, magnetic resonance, and similar techniques.
2. ***laboratory test results***: blood markers used for the diagnosis of stroke symptoms: such as thyroid tests, glucosa, C-reactive protein test, or blood protein tests.
