# Supplementary material for: Applications of Natural Language Processing for the Management of Stroke Disorders: Scoping Review
Source: JMIR Med Inform. 2023 Sep 6;11:e48693. doi: 10.2196/48693 (PMC10512117; doi:10.2196/48693)
Supplement: Multimedia Appendix 2 [file medinform_v11i1e48693_app2.docx]

**Appendix 2: Description of AI, NLP and statistical tools**

This appendix provides brief descriptions of the procedures, methods, models and software tools involved in the reviewed papers, classified in four categories (NLP, AI and general statistics, software packages, and performance metrics), and sorted by alphabetical order of their names or abbreviations within each category. Bibliographic references are numbered as in the main paper (<http://dx.doi.org/10.2196/48693>).

***NLP-specific methods and tools***

**BERT (Bidirectional Encoder Representations from Transformers)**: Language model published in 2018 by Software Engineers from Google [47].

**BOW (Bag-of-words)**: Representation of texts as a multiset of tokens (words or pseudo-words), in which each token is characterized by its frequency of occurrence in the text.

**ConText**: Subtask in information retrieval task that extends the concept of NegEx, determining whether clinical conditions mentioned in clinical reports are negated, hypothetical, historical, or experienced by someone other than the patient [49].

**Named Entity Recognition**: Information retrieval task, consisting in the classification of words contained in a text into pre-defined categories such as person names, organizations, locations, medical codes, time expressions, quantities, monetary values, percentages, etc. It can be used as a tool to work with ontologies.

**Negation extraction (NegEx)**: Subtask in information retrieval tasks, consisting in the extraction of particles like adverbs, affixes or expressions that negate or invert the meaning of other items contained in the text.

**N-gram**: Contiguous sequence of “N” items (usually words, but can be letters, phonemes, syllables, or other elements of speech) that are used in the tokenization of texts.

**Tokenization**: Segmentation of a text in a sorted array of tokens (often words, but they can be shorter or longer items), often used as a first step of NLP pipelines.

**Ontologies**: Set of concepts and categories in a subject area or domain that shows their properties and the relations between them. Used in NLP to analyze relationships between contents of the texts and pre-defined standard vocabularies, or between parts of the text or different texts according to their semantics.

**Regular expressions (REG-EXPR)**: Codified sequences of characters that define arbitrary text patterns, used in string-searching algorithms. The codes of regular expressions can represent specific characters, or groups of characters (numbers, letters, spaces, punctuation symbols, or user-defined sets of characters), as well as logical or repetition patterns.

**Systematized Nomenclature of Medicine (SNOMED)**: Computer-processable collection of medical terms which are coded with synonyms and definitions, to work with ontologies.

**Term Frequency-Inverse Document Frequency word embeddings (TF-IDF)**: Statistic that characterizes the importance of unique terms in a set of documents, and is used as an alternative of bags-of-words. The value of the TF-IDF statistic also increases proportionally to the number of times a word appears in a document, but it is offset by the number of documents in the corpus that contain the word, in order to reduce the weight of commonplace words that occur frequently in any kind of text.

**Unified Medical Language System (UMLS)**: Compendium of vocabularies of biomedical sciences with a mapping structure that allows to translate among vocabulary systems and work with ontologies.

**Web Ontology Language (OWL)**: Semantic markup language used to describe relationships and properties of objects and concepts on the internet. In NLP, they can be used to characterize terms found in the texts with rules based on ontologies.

**Word-embedding**: Representation of tokens or texts as numerical vectors of an arbitrary dimension, defined by Machine Learning techniques, where the similarity between vectors of two given words or texts is related to their semantic or lexical proximity.

***AI and general statistics***

**Classification And Regression Tree (CART)**: Variation of a Decision Tree algorithm that can handle both classification and regression tasks.

**Convolutional Neural Network (CNN)**: Artificial neural network that, instead of (or complementary to) fully connected layers of neurons, uses layers based on convolutional filters to transform the data. Often used in Deep Learning architectures to process images, or more generally data structured in N-dimensional arrays.

**Decision Tree**: Decision support tool based on a set of conditional (“if-else”) statements, interconnected in a tree-like model.

**Deep Learning (DL)**: Class of Machine Learning algorithms that use multiple layers to progressively extract higher-level features from the input, without need of hand-crafted extraction of those features.

**Gradient Boosting**: Machine Learning technique that creates high-performance models as an optimized assembly of “weaker” models, which are often Decision Trees.

**K-nearest neighbors (K-NN)**: Machine Learning algorithm used for regression and classification, in which the input is evaluated according to its proximity to the data used for training.

**Least Absolute Shrinkage and Selection Operator (LASSO)**: Type of regression modelling algorithm, which reduces the set of initial covariates to enhance the prediction accuracy and interpretability of the models.

**Logistic Regression (LR)**: Statistical model used for binary classification, which models the probability of the output being true or false through as a regression of the log-odds equivalent to that probability.

**Long-Short Term Memory (LSTM)**: Type of recurrent artificial neural network, used to process long sequences of data, in which the input of each neuron also includes its own output from previous activations, with operators (“gates”) that regulate dynamically how inputs and outputs incorporated or discarded in each step. Often used in Deep Learning architectures to process time-dependent data in general, or language in particular.

**Machine Learning (ML)**: General term used to describe algorithms to solve computational problems (regression, classification, prediction...) creating models with initially undefined parameters, which are progressively adjusted by a learning process based on sample data. They differ from other statistical models in their focus on prediction vs. inference, a greater emphasis on data, and tendency to greater complexity.

**Multi-Layer Perceptron (MLP)**: Type of artificial neural network based typically used in Deep Learning, based on stacking multiple layers of fully connected neurons that make successive transformations of the input data.

**Naïve Bayes classifier**: A technique to make probabilistic classifiers by the composition of conditional probabilities through Bayes’ theorem, assuming independence between features of the input data. Often used

**Principal Component Analysis (PCA)**: Statistical technique used to simplify the dimensionality of a data set, calculating a linear transformation of the data that concentrates their variance in the first dimensions, so that the projections of the data in higher dimensions can be omitted with little statistical variation.

**Random Forests (RF)**: Machine Learning technique that operates by constructing multiple Decision Trees at training time, and provides an output based on the ensemble of the individual outputs (either by majority or average, depending on the task).

**Support Vector Machines (SVM)**: Set of Machine Learning techniques to classify data, which creates nonlinear boundaries in the input domain transforming hyperplanes with “kernel” functions, whose parameters are learned from sample data.

***Software packages***

**BRAT Rapid Annotation Tool**: Web-based tool for collaborative annotation (<https://brat.nlplab.org/index.html>).

**CHARTextract**: Rule-based information extraction tool for Electronic Health Records (EHR) in Python and JavaScript (<https://lks-chart.github.io/CHARTextract-docs/>).

**cTAKES**: NLP tool for extraction of information from (EHR) in Java (<https://ctakes.apache.org/>).

**HD-NLP**: High Definition Natural Language Processing; pipeline developed by the University of Buffalo to search information in EHR with Ontologies [50].

**Jazzy spell checker**: Java-based spell checker tool (<https://github.com/kinow/jazzy>).

**Keras**: Library to create Machine Learning models with Tensorflow in Python (<https://keras.io/>).

**MedCAT**: Tool in Python to extract information from EHR and link it to biomedical ontologies like SNOMED and UMLS (<https://github.com/CogStack/MedCAT>).

**MedTagger**: Web-based collaborative framework for annotating medical datasets (<https://github.com/medtagger/MedTagger>).

**MetaMap**: Online tool provided by the US National Library of Medicine to map biomedical text to the UMLS Metathesaurus and discover Metathesaurus concepts referred to in text (<https://lhncbc.nlm.nih.gov/ii/tools/MetaMap.html>).

**NLTK**: Natural Language Processing toolkit for Python (<https://www.nltk.org/>)

**pyConTexT**: Package to apply the ConText tool in Python (<https://pypi.org/project/pyConTextNLP/>).

**Quanteda**: Package for managing and analyzing textual data in R (<http://quanteda.io/>)

**scikit-learn**: Library for Machine Learning algorithms in Python (<https://scikit-learn.org>)

**SemEHR**: Software architecture developed by King’s College London to search information in EHR with Ontologies [51].

**spaCy**: Framework to implement NLP pipelines in Python (<https://spacy.io/>).

**Tensorflow**: Optimized library to create Machine Learning models in C++ (<https://www.tensorflow.org/>).

**Weka**: Machine Learning toolkit in Java (<https://www.cs.waikato.ac.nz/ml/weka/>)

**XGBoost**: Multi-language package for Gradient Boosting (<https://xgboost.readthedocs.io/en/stable/index.html>)

***Performance metrics***

**Accuracy**: Ratio of correctly classified cases (true positives + true negatives) to the total number of cases that have been evaluated.

**Area Under the Curve (AUC)**: Measure of discriminative ability of a binary classifier, calculated as the integral of the ROC curve. Equivalent to the C-stastistic.

**Cohen’s kappa**: Measure of reliability based on ratios of classifications, with similar interpretation to Accuracy, but adjusted to take into account chance agreement.

**C-statistic**: Measure of reliability of a binary classifier, calculated as the integral of the ROC curve. Equivalent to the Area Under the Curve (AUC).

**F1 score**: Harmonic mean of Precision and Recall.

**Integrated Discrimination Index (IDI)**: Measure of improvement of classifier with respect to a baseline model, based on differences of the ROC [48].

**Negative Predictive Value (NPV)**: Ratio of true negatives to the total number of negative outcomes (true negatives + false negatives).

**Net Reclassification Index (NRI)**: Measure of improvement of a classifier with respect to a baseline model, based on differences of true and false positive and negative outcomes [48].

**Positive Predictive Value (PPV)**: Ratio of true positives to the total number of positive outcomes (true positives + false positives). Equivalent to Precision.

**Precision**: Ratio of true positives to the total number of positive outcomes (true positives + false positives). Equivalent to PPV.

**Recall**: Ratio of true positives to the total number of positive cases (true positives + false negatives). Equivalent to Sensitivity.

**Receiver Operating Characteristic (ROC) curve**: Graphical representation of the diagnostic ability of a binary classifier at various thresholds.

**Sensitivity**: Ratio of true positives to the total number of positive cases (true positives + false negatives). Equivalent to Recall.

**Specificity**: Ratio of true negatives to the total number of negative cases (true negatives + false positives).
